# Pulsed Radiofrequency of the Stellate Ganglion for Tinnitus: A Two‐Case Series Suggesting a Novel Approach

**DOI:** 10.1155/cria/3203716

**Published:** 2026-09-24

**Authors:** Estefanía Romero-Serrano

**Affiliations:** ^1^ Department of Anesthesiology and Pain Medicine, Hospital de Manises, Valencia, Spain, hospitalmanises.es

## Abstract

**Background:**

There is limited literature on therapies targeting the stellate ganglion for the treatment of tinnitus. Stellate ganglion interventions may modulate sympathetic tone, cerebral blood flow, and central sensory networks involved in auditory perception, providing a potential mechanism for tinnitus improvement.

**Case Presentation:**

We present two clinical cases of long‐standing tinnitus treated with pulsed radiofrequency (PRF) and local anesthetic with corticosteroids. The intensity of tinnitus decreased 2 months later, and the level of disability measured with the Tinnitus Handicap Inventory (THI) decreased from a catastrophic to a moderate score. The procedure was well tolerated, with no reported adverse events.

**Conclusions:**

These cases suggest that PRF of the stellate ganglion may represent a safe and promising therapeutic option for tinnitus. However, further study is needed to clarify its role in this indication.

## 1. Introduction

Tinnitus is a highly prevalent disease, affecting more than 740 million adults worldwide and perceived as a major problem by more than 120 million people [1]. Tinnitus is often a disabling condition that is difficult to treat. Some studies estimate that patients may incur average annual costs of approximately 565€ [2]. Its etiology [3] requires a very broad differential diagnosis since it may be related to neoplasm, vascular pathology (highlighting arteriovenous malformation or fistula), intracranial hypertension, otological pathology, chronic pain, temporomandibular joint pathology, and psychological and psychiatric disorders, among others, often without being able to establish a clear diagnosis, which leads to a poor prognosis of the disease, which in many patients follows a chronic and difficult‐to‐treat course.

In this sense, one of the therapeutic indications for the treatment of the stellate ganglion is tinnitus [4]. A bibliographic search in January 2025 (language filter: English) with the descriptors [stellate ganglion] AND [tinnitus] in PubMed yields 11 results (from 1964 to 2021) and in Cochrane 5 results. Only two clinical trials stand out that claim that treatment of the stellate ganglion can be beneficial in cases of tinnitus: blockade with local anesthetic [5] and xenon phototherapy [6]. Although the therapeutic approach to the stellate ganglion in tinnitus was described many years ago, there does not seem to be enough scientific evidence to support it, and novel technologies such as radiofrequency have not been incorporated into scientific studies, so it would be necessary to greatly expand the medical literature in this field. This paper shows two cases of patients who, after having been treated with radiofrequency in the stellate ganglion and with local anesthesia and corticosteroid, have evolved favorably 2 months later.

## 2. Clinical Cases Presentation

### 2.1. Case 1

A 63‐year‐old male patient, allergic to penicillin and suffering from arterial hypertension, Type 2 diabetes mellitus, fibromyalgia, depressive syndrome, hepatitis B virus and surgical history with appendectomy, knee meniscectomy, and carpal tunnel syndrome.

The patient was referred from Otorhinolaryngology due to tinnitus of approximately 5 years’ duration. Tinnitus is perceived bilaterally and intermittently and without any associated auditory pathology or hearing loss although there are isolated episodes of vertigo (Table 1).

**TABLE 1:** Demographic and clinical characteristics of the patients.

Characteristic	Case 1	Case 2
Sex	Male	Female
Age (years)	63	82
Academic level	Primary education	Primary education
Bilateral tinnitus	Yes	Yes

The patient was assessed using the Tinnitus Handicap Inventory (THI) with 78 points (catastrophic level of disability due to tinnitus). In addition, before the technique, the patient reported perceiving a tinnitus intensity of 8/10 in the right ear (RE) and 8/10 in the left ear (LE).

Intervention was performed on the left stellate ganglion, identifying the structure with fluoroscopy and ultrasound. The procedure was performed at the level of C6, which provides optimal visualization of the sympathetic chain and minimizes the risk of vascular or pleural complications. The patient was subjected to pulsed radiofrequency (PRF) (65 V, 42°C, 2 Hz, 20‐ms pulse width for 360 s) using a temperature‐controlled radiofrequency generator (CR‐10; Cardiva, Spain), 22‐gauge, 10‐cm cannula with a 5‐mm active tip. Subsequently, 5 mL of 0.25% levobupivacaine and 4‐mg dexamethasone were administered.

**FIGURE 1:**
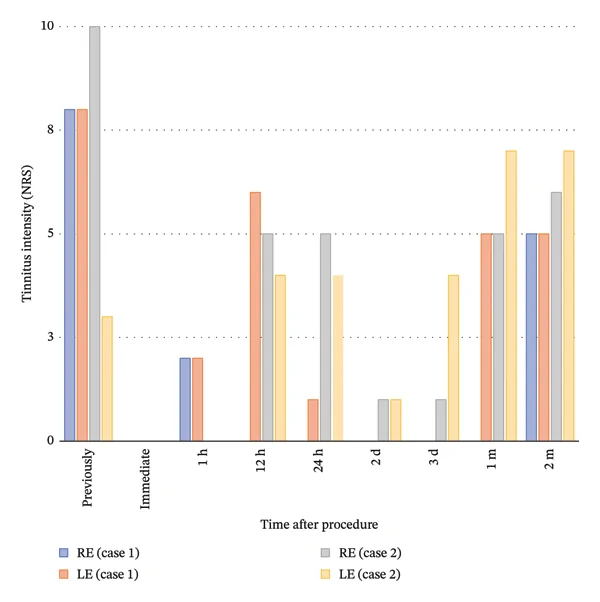
Evolution of tinnitus intensity with numerical rating score (NRS). RE: right ear; LE: left ear. Times: previously, immediate, 1 h later (1h), 12 h later (12h), 24 h later (24h), 2 days later (2d), 3 days later (3d), 1 month (1m), and 2 months later (2m). Abbreviations: h, hour(s); d, day(s); m, month(s); RE, right ear; LE, left ear; NRS, numerical rating scale.

**FIGURE 2:**
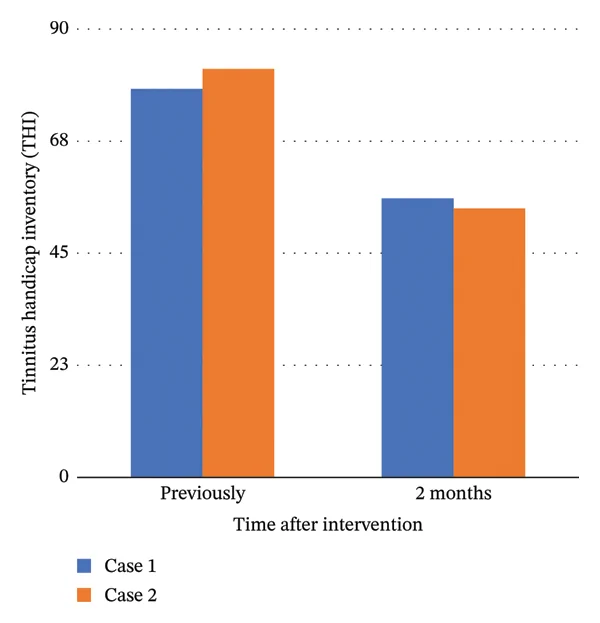
Evolution of the Tinnitus Handicap Inventory (THI).

Finally, 2 months later, THI was 56 points (moderate level of disability due to tinnitus), and tinnitus intensity scores were 5/10 in both ears. No episodes of vertigo were reported during the follow‐up period. Patient satisfaction with the procedure was estimated at approximately 50%, based on a direct numerical rating reported during the follow‐up. No formal validated satisfaction scale was administered (Figures 1 and 2). After the intervention, tinnitus intensity decreased early during follow‐up and remained lower than baseline at 2 months although with partial loss of the initial benefit over time.

### 2.2. Case 2

An 82‐year‐old woman suffering from arterial hypertension, Type 2 diabetes mellitus, dyslipidemia, fibromyalgia, depressive syndrome, hypertrophic cardiomyopathy and with surgical history carrier of a stent in the anterior descending coronary artery (2023), knee prosthesis, and blepharoplasty. Antiplatelet therapy with acetylsalicylic acid 100 mg/day and clopidogrel 75 mg/day was performed. Given the patient’s dual antiplatelet therapy, clopidogrel was discontinued 5 days before the procedure, while acetylsalicylic acid 100 mg/day was continued. Clopidogrel was restarted 24 h after the intervention without complications.

The patient was referred from Otorhinolaryngology due to tinnitus of 32 years’ duration, perceived bilaterally and intermittently, with no other associated hearing pathology or hearing loss (Table 1).

THI was previously evaluated with 82 points (catastrophic level of disability due to tinnitus). Scores of tinnitus intensity prior to the technique were 10/10 (RE) and 8/10 (LE).

The same interventional technique was performed on the right stellate ganglion at the level of C6 under ultrasound and fluoroscopic guidance. Two months later, the patient had a THI of 54 points (moderate level of tinnitus disability) and reported tinnitus intensity of 6/10 (RE) and 7/10 (LE). The patient reported a subjective satisfaction level of approximately 60% during follow‐up, based on a direct numerical rating rather than a validated scale (Figures 1 and 2). A similar response pattern was observed, with early reduction in tinnitus intensity after the procedure and partial recurrence over follow‐up, while still remaining improved compared with baseline at 2 months.

## 3. Discussion

Matoba et al. [5] described how local anesthetics have been used for the treatment of tinnitus since the report of Lewy [7] in 1937. Local anesthetics provide vasodilatory action due to the suppression of sympathetic nerve activity. Blockade of the stellate ganglion produces vasodilation and increases blood flow in the inner ear, reducing or eliminating tinnitus in patients.

An association between tinnitus and chronic pain is being studied by several authors. Interestingly, both patients also had fibromyalgia, which has been studied in association with tinnitus [8, 9]. A multidisciplinary approach would certainly be necessary, considering treatments such as white noise (from hearing aids to applications for technological devices accessible to the entire population), treatment of temporomandibular joint pathology, or psychological therapy.

Neither patient had a documented diagnosis of peripheral neuropathy, small‐fiber neuropathy, or mononeuropathy. However, these conditions were not systematically assessed and, therefore, could not be excluded or analyzed as potential contributing factors, including possible neuropathic involvement of the auditory system. This may represent a relevant variable for future studies.

The mechanism by which PRF of the stellate ganglion may improve tinnitus remains uncertain. Beyond the local vasodilatory effect mediated by sympathetic blockade, PRF may exert a broader neuromodulatory influence on central networks involved in sympathetic tone and sensory perception. Experimental studies have shown that PRF can modulate microglial activation, synaptic plasticity, and cytokine release in central pain pathways, suggesting that its effects extend beyond peripheral structures (Chua et al.) [10]. Functional neuroimaging studies have demonstrated that unilateral stellate ganglion interventions induce bilateral cortical changes, particularly in somatosensory, thalamic, and limbic regions (Kim et al.) [11], while recent human data confirm modulation of cerebral blood flow and neuroinflammatory markers following stellate blockade (Kostadinov et al.) [12]. These findings support a potential central mechanism underlying the bilateral tinnitus improvement observed after unilateral PRF in our patients.

Regarding the treatment of the stellate ganglion in tinnitus, the available clinical trials employ blockade with local anesthesia [5, 7], xenon phototherapy [6], but no scientific evidence has been found for radiofrequency ablation results in tinnitus. Notably, there are comparative studies between stellate ganglion ablation with ethanol or thermal radiofrequency [13] with other therapeutic indications, with superior results using radiofrequency. Our findings are consistent with these observations, as both of our patients showed improvement following stellate ganglion modulation. Matoba et al. [5] reported symptomatic relief after local anesthetic blockade, while Shimizu et al. [6] demonstrated benefit with xenon phototherapy. In contrast, our cases appear to be the first to describe PRF of the stellate ganglion for tinnitus, suggesting that neuromodulatory interventions may extend therapeutic possibilities beyond transient sympathetic blockade.

The anatomical complexity of the cervical sympathetic chain, including the presence of variable accessory fibers such as the Kuntz nerves and extensive interconnections with adjacent neural structures, may limit the efficacy of neurodestructive techniques based on complete interruption of sympathetic transmission. In this context, PRF, which exerts its effects through neuromodulation rather than thermal lesioning, may be less dependent on precise anatomical interruption. By modulating neural signaling without requiring complete fiber ablation, PRF could theoretically overcome anatomical redundancy within the stellate ganglion region and provide a more sustained therapeutic effect.

Beyond hemodynamic and neuromodulatory mechanisms, stellate ganglion blockade has been increasingly associated with immunomodulatory effects. Experimental and clinical studies have demonstrated reductions in proinflammatory cytokines—particularly interleukin‐6 (IL‐6)—following stellate ganglion block in inflammatory and autoimmune conditions, including ulcerative colitis and systemic inflammatory states [14]. These findings suggest that modulation of the cervical sympathetic chain may influence peripheral and central inflammatory signaling.

Recent evidence has also highlighted a potential link between tinnitus and systemic inflammation [15]. Tanaka et al. reported a significant association between tinnitus severity and inflammatory biomarkers in older adults, supporting the hypothesis that tinnitus may, at least in part, be influenced by low‐grade inflammatory processes. In this context, the immunomodulatory effects of stellate ganglion interventions may represent an additional mechanism contributing to tinnitus improvement, complementing vascular and neuromodulatory pathways.

Additionally, thermal neurodestructive techniques applied to the stellate ganglion may show variable durability due to anatomical redundancy of the sympathetic chain, including longitudinal interconnecting fibers (Dretz fibers) that allow conduction to persist despite focal lesions. Because PRF does not rely on complete anatomical interruption, its therapeutic potential is theoretically less dependent on these anatomical variations. This rationale supported the decision to proceed directly with PRF.

Some authors have suggested functional lateralization of the stellate ganglion, with the right side more closely related to limbic and central autonomic modulation and the left side more strongly associated with cardiac sympathetic regulation. However, current evidence is insufficient to determine whether laterality has a clinically relevant impact on tinnitus outcomes, and this remains an area for further investigation.

The THI is a validated and widely adopted measure of tinnitus‐related handicap, with high internal consistency and test–retest reliability. Its use in our cases provides a standardized assessment of patient‐reported disability over time [16].

Levobupivacaine was chosen for its intermediate duration of action and its improved cardiovascular and neurologic safety profile compared with racemic bupivacaine, particularly relevant in procedures involving the cervical sympathetic chain where unintentional intravascular spread may occur. Dexamethasone was added to the injectate to extend the duration of the analgesic and sympathetic‐modulating effects, a practice commonly used in stellate ganglion interventions.

A relevant limitation of these case reports is the inability to clearly distinguish the relative contribution of PRF, the local anesthetic injection, and dexamethasone to the observed clinical improvement. Although previous literature has described prolonged symptomatic relief following stellate ganglion block with local anesthetic alone, dexamethasone may also have contributed to the early reduction in tinnitus intensity and the subsequent gradual waning of benefit over time. Therefore, these cases cannot determine whether the observed effect is attributable primarily to PRF or to the combined effect of stellate ganglion modulation with local anesthetic and steroid. Nevertheless, the sustained improvement observed at 2 months suggests that mechanisms beyond transient sympathetic blockade may have contributed to the clinical outcome. Controlled studies comparing PRF plus injectate versus stellate ganglion block with local anesthetic and steroid alone would be necessary to clarify the specific contribution of PRF.

From an immunological perspective, some authors have suggested that bilateral stellate ganglion block or multilevel cervical approaches (e.g., C6 and C4) may enhance systemic sympathetic and inflammatory modulation by achieving a broader field block. In contrast, PRF is inherently more focal, targeting localized neuromodulatory effects. While bilateral or multilevel stellate ganglion interventions may theoretically provide additional immunological benefits, their role in tinnitus management remains insufficiently studied.

Despite the patients’ cardiovascular comorbidities, the procedures were well tolerated under ultrasound guidance and standard monitoring, without hemodynamic instability or arrhythmias, supporting the safety of PRF in carefully selected cases. This therapy may represent a promising option in selected patients with refractory tinnitus. It is necessary to increase the medical literature on the subject and continue conducting clinical trials to increase scientific evidence. Future prospective studies may evaluate whether bilateral or multilevel approaches offer superior or more sustained outcomes compared with unilateral techniques.

## 4. Conclusions

These case reports show two patients that present arterial hypertension, Type 2 diabetes mellitus, and chronic pain (fibromyalgia). In addition, in one of the cases, there was cardiovascular disease (coronary artery disease treated with a stent). After treatment with PRF on the stellate ganglion, both patients had a favorable evolution, with a decrease of the tinnitus intensity scores (at least 30%), a decrease of the THI score (from catastrophic to moderate level). In one case report, the tinnitus suffered on the contralateral level was perceived as worsened, which reinforces the perception of improvement on the side undergoing treatment. Patients felt satisfied with the treatment, and no adverse events were reported. These cases suggest that pulsed radiofrequency of the stellate ganglion may represent a safe and promising therapeutic option for tinnitus. Future studies should include prospective controlled trials comparing PRF plus stellate block with sham interventions, longer follow‐up, and neuroimaging correlates to elucidate underlying mechanisms.

## Author Contributions

Estefanía Romero‐Serrano contributed to the conception, design, data collection, analysis, and writing of the manuscript.

## Funding

This research received no external funding.

## Disclosure

The author has approved the final version of the manuscript.

## Consent

Written informed consent was obtained from all patients for the publication of these case reports and any accompanying images.

## Conflicts of Interest

The author declares no conflicts of interest.

## Data Availability

All relevant data supporting the findings of this study are included within the article.

## References

[1] Jarach C. M. , Lugo A. , Scala M. et al., Global Prevalence and Incidence of Tinnitus: A Systematic Review and Meta-Analysis, JAMA Neurology. (September 2022) 79, no. 9, 888–900, 10.1001/jamaneurol.2022.2189. PMC9361184 35939312

[2] Jarach C. M. , Karydou K. , Trochidis I. et al., The Out-of-Pocket Expenses of People With Tinnitus in Europe, Journal of Epidemiology. (November 2024) 34, no. 11, 515–525, 10.2188/jea.JE20230358. PMC11464849 38797674

[3] Wang H. , Stern J. I. , Robertson C. E. , and Chiang C. C. , Pulsatile Tinnitus: Differential Diagnosis and Approach to Management, Current Pain and Headache Reports. (August 2024) 28, no. 8, 815–824, 10.1007/s11916-024-01263-1. 38842617

[4] Freire E. and Camba M. A. , Técnicas e Indicaciones del Bloqueo del Ganglio Estrellado Para el Tratamiento del Dolor, Revista de la Sociedad Española del Dolor. (June 2002) 9, 328–337.

[5] Matoba T. , Noguchi I. , Noguchi H. , and Sakurai T. , Stellate Ganglion Block for the Relief of Tinnitus in Vibration Disease, Kurume Medical Journal. (1984) 31, no. 4, 295–300, 10.2739/kurumemedj.31.295. 6543892

[6] Shimizu M. , Matsuzuka T. , Matsumi F. , Ogawa H. , and Murono S. , Change of Tinnitus With Xenon Phototherapy of the Stellate Ganglion, Photomedicine and Laser Surgery. (September 2018) 36, no. 9, 468–471, 10.1089/pho.2017.4431. 30096265

[7] Lewy R. B. , Treatment of Tinnitus Atrium by Intravenous Use of Local Anesthetic Agents, Archives of Otolaryngology. (1937) 25, no. 2, 178–183, 10.1001/archotol.1937.00650010200005.

[8] Puri B. K. and Lee G. S. , Tinnitus in Fibromyalgia, Puerto Rico Health Sciences Journal. (December 2021) 40, no. 4, 188–191. 35077079

[9] Cil Ö. Ç. , Zateri C. , Güçlü O. , Oymak S. , and Tezcan E. , The Effect of Fibromyalgia Treatment on Tinnitus, American Journal of Otolaryngology. (May 2020) 41, no. 3, 10.1016/j.amjoto.2020.102390. 31926598

[10] Chua N. H. , Vissers K. C. , and Sluijter M. E. , Pulsed Radiofrequency Treatment in Interventional Pain Management: Mechanisms and Potential Indications—A Review, Acta Neurochirurgica. (2011) 153, no. 4, 763–771, 10.1007/s00701-010-0881-5. PMC3059755 21116663

[11] Kim D. Y. , Park C. A. , Chung R. K. , and Kang C. K. , Effect of Stellate Ganglion Block on the Cerebral Cortex: A Functional Magnetic Resonance Imaging Study, Applied Magnetic Resonance. (2016) 47, no. 1, 101–109, 10.1007/s00723-015-0735-7.

[12] Kostadinov I. , Avsenik J. , Osredkar J. , Jerin A. , and Gradisek P. , Effect of Stellate Ganglion Block on Brain Hemodynamics and the Inflammatory Response in Moderate and Severe Traumatic Brain Injury: A Pilot Study, Regional Anesthesia and Pain Medicine. (June 2025) 51, no. 6, 687–694, 10.1136/rapm-2024-106185. 39971387

[13] Thabet T. S. and Khedr S. A. , Stellate Ganglion Destruction With Alcohol Versus Thermal Ablation for Chronic Post-Mastectomy Pain: A Randomized Trial, Pain Physician. (February 2024) 27, no. 2, E231–E238. 38324788

[14] Dai S. , Ji J. , Li R. , Gao L. , and He X. , Stellate Ganglion Block Attenuates LPS-Induced Acute Lung Injury by Activating Sirt3 Regulation of Oxidative Stress and Inflammation, Biomedicines. (May 2024) 12, no. 6, 10.3390/biomedicines12061148. PMC11200983 38927355

[15] Tanaka L. S. , de Moraes Marchiori L. L. , Ciquinato D. D. et al., Inflammatory Biomarkers and Tinnitus in Older Adults, Noise and Health. (2024) 26, no. 123, 535–542. 10.4103/nah.nah_39_23 PMC11813249 39787555

[16] Newman C. W. , Jacobson G. P. , and Spitzer J. B. , Development of the Tinnitus Handicap Inventory, Archives of Otolaryngology—Head and Neck Surgery. (1996) 122, no. 2, 143–148, 10.1001/archotol.1996.01890140029007. 8630207

